# Supplementary material for: Graphene-Based Membranes for Water Desalination: A Literature Review and Content Analysis
Source: Polymers (Basel). 2022 Oct 10;14(19):4246. doi: 10.3390/polym14194246 (PMC9571434; doi:10.3390/polym14194246)
Supplement: Supplementary file 1 [file polymers-14-04246-s001.zip › polymers-1887029-information.pdf]

Supplementary Information

Graphene-Based Membranes for Water Desalination: A Literature Review and Content Analysis

Yexin Dai [1], Miao Liu [1], Jingyu Li 1, Ning Kang [1], Afaque Ahmed [1], Yanping Zong [2], Jianbo Tu [2], Yanzhen Chen [2], Pingping Zhang [3] and Xianhua Liu [1,*]

1 School of Environmental Science and Engineering, Tianjin University, Tianjin 300354, China

2 Tianjin Marine Environmental Center Station, Ministry of Natural Resources, Tianjin 300450, China

3 College of Food Science and Engineering, Tianjin Agricultural University, Tianjin 300384, China

* Correspondence: lxh@tju.edu.cn; Tel.: +86-22-85356239

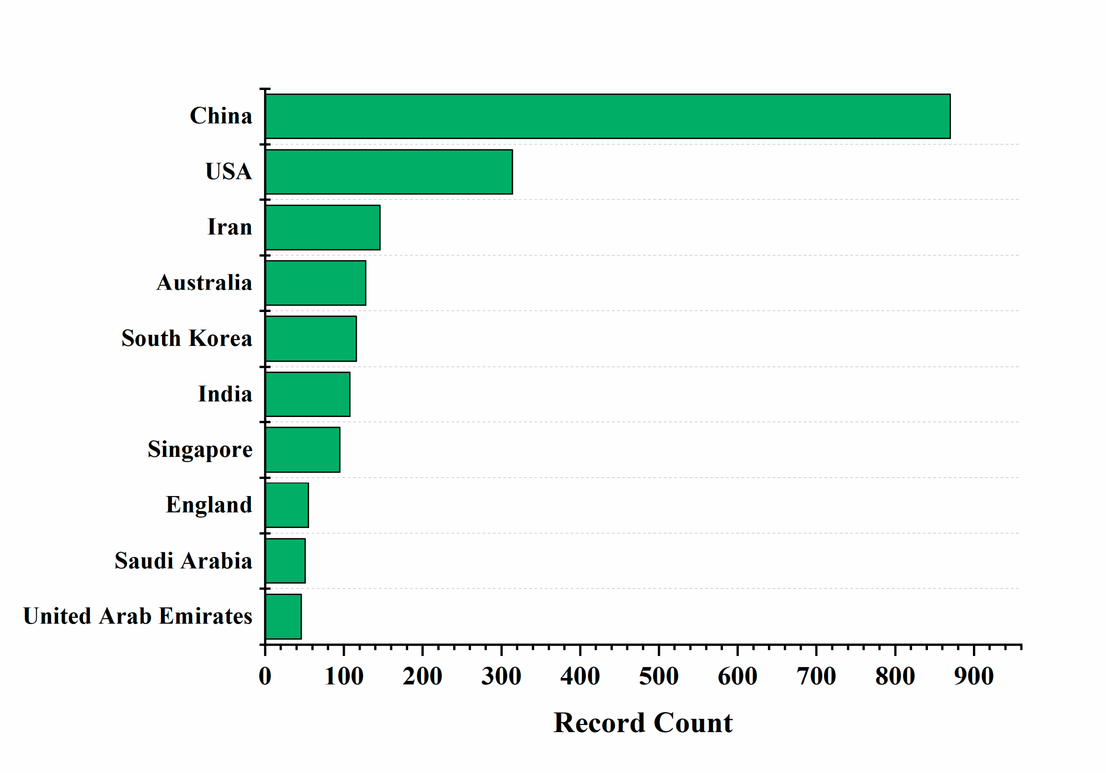

Figure S1. Top 10 Countries of highest number of publications

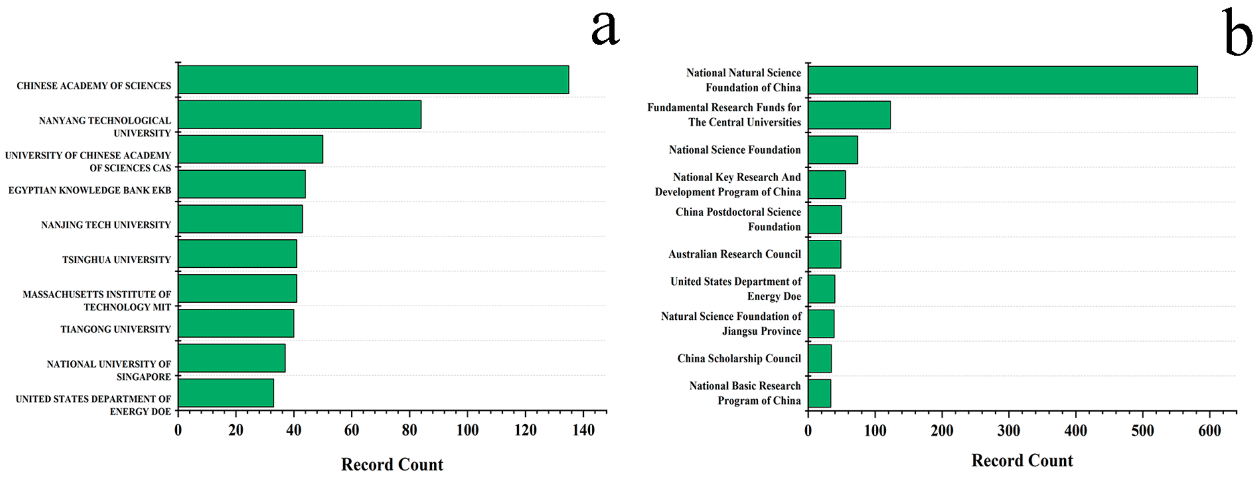

Figure S2. (a) Top 10 affiliated institutes, (b) Top 10 funding sponsors.

Table S1. Top 10 countries with highest number of publications

No.	Country	Documents	Citations	Average Citations
1	China	870	13767	15.82
2	USA	314	3786	12.05
3	Iran	146	1598	10.94
4	Australia	128	1328	10.37
5	South Korea	116	1496	12.89
6	India	108	1832	16.96
7	Singapore	95	872	9.17
8	England	55	879	15.98
9	Saudi Arabia	51	778	15.25
10	United Arab Emirates	46	308	6.69

Table S2. Top 15 journals with highest number of publications.

Rank	Journals	Publications	Citations	Total Link Strength	IF2021
1	Desalination	167	7715	2277	11.21
2	Journal of Membrane Science	156	6933	1902	10.53
3	Separation and Purification Technology	73	1311	702	9.13
4	Journal of Materials Chemistry A	66	4902	1032	14.51
5	ACS Applied Materials Interfaces	65	3141	919	10.38
6	Carbon	43	1749	721	11.30
7	Membranes	38	289	297	4.56
8	Chemical Engineering Journal	35	1148	362	16.74
9	RSC Advances	35	900	337	4.03
10	Desalination and Water Treatment	29	151	215	1.27
11	Physical Chemistry Chemical Physics	27	573	332	3.94
12	Nanoscale	23	1259	378	8.30
13	ACS Nano	22	2520	460	18.02
14	Applied Surface Science	21	701	303	7.392
15	Journal of Physical Chemistry C	21	412	240	4.177

Table S3. Top 15 research categories with highest number of publications.

**No.**	**Subject Category**	**Publications**	% of 1,739
1	Engineering	733	42.151
2	Chemistry	678	38.988
3	Materials Science	622	35.768
4	Science Technology Other Topics	393	22.599
5	Physics	273	15.699
6	Polymer Science	246	14.146
7	Water Resources	244	14.031
8	Energy Fuels	124	7.131
9	Environmental Sciences Ecology	122	7.016
10	Biochemistry Molecular Biology	53	3.048
11	Computer Science	13	0.748
12	Electrochemistry	13	0.748
13	Biotechnology Applied Microbiology	9	0.518
14	Mechanics	9	0.518
15	Crystallography	8	0.46

Table S4. Frequency of top 15 author keywords

Id	Keyword	Occurrences	Total Link Strength
1	Desalination	326	315
2	Graphene Oxide	244	268
3	Graphene	119	135
4	Membrane	118	170
5	Reverse Osmosis	84	121
6	Forward Osmosis	83	87
7	Nanofiltration	73	89
8	Molecular Dynamics	67	82
9	Water desalination	63	36
10	Membranes	51	56
11	Water purification	51	50
12	Membrane distillation	46	47
13	Water treatment	45	51
14	Interfacial polymerization	45	38
15	Antifouling	39	43
